# Evidence that inflammation promotes estradiol synthesis in human cerebellum during early childhood

**DOI:** 10.1038/s41398-018-0363-8

**Published:** 2019-01-31

**Authors:** Christopher L. Wright, Jessica H. Hoffman, Margaret M. McCarthy

**Affiliations:** Department of Pharmacology and Program in Neuroscience, University of Maryland School of Medicine, Baltimore, MD 21201 USA

## Abstract

Discovering and characterizing critical and sensitive periods in brain development is essential for unraveling the myriad variables that impact disease risk. In previous work, we identified a critical period in cerebellar development in the rat that depends upon an intrinsic gene expression program and links increased prostaglandin production to local estradiol synthesis by stimulating Cyp19a, the estradiol synthetic enzyme, aromatase. This intrinsic critical period is sensitive to disruption by either inflammation or administration of cyclooxygenase (COX) inhibitors, ultimately impacting Purkinje cell dendritic growth. In a first step towards determining if a similar sensitive period exists in humans, the same gene expression profile was characterized in post-mortem cerebellar tissue of 58 children aged 0 to 9 years. Subjects were categorized as experiencing inflammation or not at the time of death. In individuals experiencing inflammation and over 1 year of age, there was a significant increase in the messenger RNA (mRNA) of the COX-1 and COX-2 enzymes and this strongly correlated with mRNA levels of aromatase. A step-wise linear model accounted for 94% of the variance in aromatase mRNA levels by co-variance with the COX enzymes, prostaglandin E2 synthase and other inflammatory mediators (Toll-like receptor 4), and Purkinje cell markers (calbindin, estrogen receptor 2). The influence of inflammation on these measures was not seen in subjects younger than 1 year. These data suggest a sensitive period to inflammation in the human cerebellum begins at about 1 year of age and may provide insight into sources of vulnerability of very young children to either inflammation or drugs designed to treat it.

## Introduction

The cerebellum is one of the first brain structures to emerge and one of the last to fully mature, at least in part because of its reciprocal closed-loop circuitry with multiple cortical regions^1^. In adulthood, the cerebellum is characterized by its role in intrinsic motor learning^2,3^, but is gaining increasing appreciation for its role early in life to shape and refine neocortical circuits for affect and cognition^4^. Children treated surgically for cerebellar tumors often experience symptoms of cerebellar cognitive syndrome, including poor decision making and planning, decreased working memory, poor speech generation, impaired visuospatial reasoning, and irritability compared to normative data^5–7^. The constellation of effects depends on which hemisphere is involved, inclusion of the vermis, and the age of surgical resection. Damage to the cerebellum in infancy is one risk factor among many contributing to whether a child is diagnosed with autism spectrum disorder (ASD), and is particularly associated with perseverative behaviors and inability to perceive or match another’s emotions^8–10^. Pathologies of the cerebellum are also strongly associated with schizophrenia^4,11^, which is increasingly being viewed as a disorder with origins in development^12–16^.

Prostaglandin E2 (PGE2) is an eicosanoid that among its other physiological roles induces a fever^17^. PGE2 is produced from arachidonic acid (AA) by two enzymes acting sequentially. First, the cyclooxygenase enzymes COX-1 and COX-2 convert AA to prostaglandin H2. Then, prostaglandin E synthase (PGES) produces PGE2. In the rodent brain, most AA is synthesized from the endocannabinoid, 2-arachidonoylglycerol, by monoacylglycerol lipase (MAG lipase), but this has not been validated in the human^18^.

Estradiol is considered a sex steroid hormone made in the gonads, but there is an increasing appreciation for local synthesis in the brain, particularly in humans^19,20^. Both isoforms of the estrogen receptor (ER) are expressed by cerebellar neurons, with ERα (*Esr1*) notable for much higher expression early in development and restriction to Purkinje cells compared to ERβ (estrogen receptor 2 (*Esr2*)). The aromatase gene (*Cyp19a*) is also expressed by developing Purkinje cells, suggesting cell autonomous developmental regulation by estradiol^21^.

The laboratory rat provides a model for identifying previously unknown sensitive windows of vulnerability to acute events, such as infection, as well as sources of sensitivity to ongoing inflammation. We have identified a neonatal sensitive period during which the cerebellum is susceptible to inflammation and mimetics of infection, resulting in impaired Purkinje neuron development. Either direct administration of the proinflammatory prostaglandin PGE2 into the cerebellum or peripheral administration of the inflammatory-inducing agent lipopolysaccharides stunts Purkinje neuron dendritic development^22,23^. Remarkably, the deleterious effects of inflammation are only manifest if the exposure occurs during the second postnatal week of life. The same treatments during the first or third week are completely without impact. The sensitive period is marked by the activation of the steroidogenic enzyme, aromatase (Cyp19a), which aromatizes androgen precursors into estrogens. Endogenous estradiol is elevated in the 2-week-old cerebellum, suggesting a normal role in maturation, but if increased in response to inflammation or direct administration, Purkinje neuron development is stunted. The sensitive period is closed at the end of the 2nd week by a precipitous drop in the expression of the *Cyp19a* gene, as well as *Esr1*. Thus, even though inflammation may induce the production of prostaglandin, without the increase in estradiol production and transduction, there are no deleterious consequences^24^.

As a first step in translating these findings to humans, we asked whether a similar coupling of inflammation to a cerebellar PGE2–estradiol pathway exists in the newborn and early childhood human cerebellum. Toward that end, we obtained samples of post-mortem human cerebellum and quantified messenger RNA (mRNA) for the components of the PGE2–estradiol synthesis pathway as well as other markers of inflammation. We predicted that: (1) the enzymes producing PGE2 would be up-regulated in individuals whose medical records indicate signs of infection or inflammation at or near death, (2) that the enzyme aromatase would also be up-regulated with infection or inflammation, and (3) that expression of enzymes producing PGE2 and receptors signaling for PGE2 would correlate with aromatase expression during inflammation, but would not correlate in expression in individuals not experiencing inflammation. Because the medical records associated with young children are limited, we also sought to confirm inflammatory status by measuring mRNA for Toll-like receptor 4 (TLR4) in the brain samples, a reliable indicator of inflammation^25^

## Materials and methods

### Selection of human cerebellar samples

The demographics of all subjects is shown in Table 1. Human tissue was obtained from the University of Maryland Brain and Tissue Bank, which expunges medical records of any individual identifiers but provides a summary of medical disposition for each individual. Samples were preselected by the UM Brain and Tissue Bank as “controls” for comparison to individuals who were previously diagnosed with a disability or disease. Every sample that met this criteria was used for a total of 58 samples of fresh-frozen cerebellum evenly split by sex, of which 21 were younger than 1 year of age. Of those, 22 were indicated as being from the lateral cerebellum, 23 from the posterior vermis, 10 were labeled generic vermis, and 3 were labeled generic cerebellum (we confirmed macroscopic cerebellar morphology). This sample size was considered adequate based on other studies involving gene expression in human post-mortem brain tissue, which used sample sizes the same or smaller than used here and detected significant differences between groups^26–28^. An investigator unaware of our experimental predictions and not involved in this study reviewed the limited medical records available and classified each case as “inflammation” if subjects either: (1) had a cause of death indicated or exacerbated by infection, asthma, asphyxia, or inflammatory tumors, or (2) were treated with antibiotics or non-steroidal anti-inflammatory drugs around death. If none of these were present subjects were classified as “none.” Tissue pH, time in freezer, post-mortem interval (PMI), and, in some cases, Aligent RNA integrity number (RIN) were recorded

**Table 1:** Demographics and covariates of human samples $\left( {\overline {\mathrm{X}} :{\mathrm{mean}},\;{\mathrm{\sigma }}:{\mathrm{standard}}\;{\mathrm{deviation}},\;3 - {\mathrm{WAY}}\;{\mathrm{ANOVAs}}} \right)$

Variable	Less than 365 days old		Greater than 365 days old	
	Inflammation	None	Inflammation	None
Age (days)	$\overline X = 154,\sigma = 95$	$\overline X = 87,\sigma = 74$	$\overline X = 1086,\sigma = 773$	$\overline X = 1451,\sigma = 923$
PMI	$\overline X = 19,\sigma = 7.05$	$\overline X = 21.6,\sigma = 8.2$	$\overline X = 17.6,\sigma = 6.7$	$\overline X = 21.1,\sigma = 10.2$
Sex (F:M)	4:4	6:7	9:8	5:15
Time tissue remained frozen (days)	$\overline X = 4426,\sigma = 1400$	$\bar X = 4292,\sigma = 1734$	$\bar X = 3610,\sigma = 1894$	$\bar X = 3476,\sigma = 2258$
RIN	$\bar X = 7.7,\sigma = 1.44$	$\bar X = 7.9,\sigma = 1.04$	$\bar X = 8,\sigma = 1.10$	$\bar X = 8.1,\sigma = 1.05$
pH	$\bar X = 5.61,\sigma = 0.32$	$\bar X = 5.64,\sigma = 0.38$	$\bar X = 5.57,\sigma = 0.27$	$\bar X = 5.82,\sigma = 0.54$
Manner of death	8 infection	4 congenital defect, 7 accident, 1 SID, 1 unknown	6 infection, 3 asphyxia complicated by asthma, 3 asthma attacks, 1 multiple including infection, 1 intussusception of bowel, 1 inflammatory myofibroblastic tumor with T cell infiltrates, 1 cardiac arrest complicated by H1N1	17 Accident (MVA or asphyxia), 1 cardiac failure, 1 respiratory failure with multisystem organ failure, 1 congenital defect
Medications		Tylenol, Amoxicillin		Cardec

*PMI* post-mortem interval, *MVA* motor vehicle accident, *RIN* RNA integrity number, *SID* sudden infant death

### RNA extraction

Preparation of RNA from human tissue utilized the protocol for fatty tissues from the RNeasy Handbook for Mini Kit (Cat. No. 74106, Qiagen) using 70–100 mg of pulverized human cerebellum tissue following the manufacturer's protocol.

### Creation of cDNA by reverse transcription

Single-stranded complementary DNA (cDNA) was generated from extracted RNA using the ABI High Capacity cDNA Reverse Transcription Kit #4368814 (Foster City, CA, USA). In brief, 1 μg RNA was suspended in 10 μl water to which reverse transcription reagents were added in the following volumes: 2.0 μl of 10× RT buffer, 0.8 μl of 25× dNTP mix, 2.0 μl of 10× RT random primers, 1.0 μl of Multiscribe Reverse Transcriptase, 1.0 μl of RNase inhibitor, and 3.2 μl of water. The reaction was carried out as follows: 10 min primer incubation at 25 °C, 120 min extension at 37 °C, and 5 min RT inactivation at 85 °C. cDNA was stored at −20 °C until use.

### Primer design

Unless otherwise mentioned, primers were designed using Primer Express from Applied Biosystems (Table S1). The primers used for each gene were common to all variants for that gene and thus potentially amplified all known and predicted variants together.

### Relative real-time PCR

We utilized a SYBR green relative quantification method with a standard curve. Each of the samples were diluted 1:30 in water before being loaded into the quantitative PCR. Samples and standards (1:3 dilutions of a stock comprising equal amounts of RNA from each sample) were run in triplicate on a 384-well plate, using a Thermo Fisher Applied Biosystems ViiA7 with 384-well block (Grand Island, NY, USA). The cycle threshold of each unknown sample was compared against the standard curve to quantify the amount of starting material in genomic equivalents (GEs). The starting material of the 1:1 standard was arbitrarily defined as 1 GE. For human tissue, we ran five different reference genes or ribosomal RNAs (rRNAs): (1) GAPDH, (2) TATA Box Protein, (3) rpl-30, (4) YWHAZ, and (5) 18S. We excluded GAPDH because its quantities dropped between 2 and 6 months of age. Binned within specific ages, TATA Box Protein, rpl-30, and YWHAZ all changed developmentally in tandem and the relative changes were within 10% of one another, whereas relative changes in 18S were not confined to this 10% cutoff. The GEs of TATA box protein, rpl-30, and YWHAZ were averaged and then this conglomerate measure was used for normalization for each gene of interest to control for amount of DNA loaded. To compare relative expression of two separate genes, samples for both genes were run on the same plate. We set identical thresholds of fluorescence for both genes and using the standard curve (diluted as follows: 1:1. 1:3, 1:9, 1:27, 1:81, 1:243), we ensured the efficiencies of each were equivalent and near 100%. We then compared the differences in the threshold cycles for each point in the two standard curves using the delta-delta Ct equation to calculate which gene was less abundant and by how much (a relative amount). All results are expressed as a ratio of the target gene GE over corresponding reference gene(s) measure.

### Statistics

PMI, brain pH, and time tissue remained frozen (TTRF) were used as covariates in the three-way multivariate analysis of variances (MANOVAs) for sex, age, and inflammation. The variance in *PTGS2* (COX-2) and *CYP19A1* (aromatase) was 10-fold higher than the other genes, so these were subjected to separate three-way ANOVAs still applying the above covariates. We had Aligent RIN for a subset of samples, but not enough to use it as a covariate in omnibus analyses. We regressed the expression of each gene against available RINs to ensure that there was no significant regression. After Omnibus analysis, we collapsed the data where there was not an effect of one of the factors at some level. We then performed Tukey’s post hoc analysis when the reduction resulted in more than two groups.

A priori, we chose to perform linear regressions for mRNA for *CYP19A* vs. *PTGS2*, *PTGS1*, and *PGES-1* mRNA across all ages. However, the MANOVA results demonstrated an interaction between age and inflammation for *CYP19A1*. Accordingly, we subjected those ages in which inflammation had an effect to regression analyses. Regressions for *CYP19A1* vs. individual genes were conducted for linear and exponential best fits with *p* < 0.05. The regressions best fit exponential curves and this allowed for automatic step-wise linear modeling using the natural logarithm of *CYP19A1* (to model CYP19A1 exponentially but generate a linear equation) and included all 20 genes measured using the following criteria: a 95% confidence level, a forward step-wise model using criteria for entry or removal of a variable based upon the Akaike information criterion for small sample size (small being relative to <100–1000s), and a significance cutoff for retaining a variable of 0.1 or less. In this way, each gene was tested for how well it models the data in relation to the other variables. The Akaike information is used to tell the algorithm controlling the model when to stop incorporating genes to avoid overfitting.

All comparisons included sex as a variable, with 24 of the 58 subjects being females.

## Results

There were no significant main effects of sex detected for any outcomes and only one significant interaction (see below).

*CYP19A1* and *PTGS2* mRNA content did not co-vary with post-mortem interval (PMI), brain pH, or TTRF. *ESR2* (ER-β), *PTGER3* (EP3), and *TLR4-v1* and *TLR4-v3* mRNA levels co-varied with PMI, but PMI did not vary across sex, age, or inflammation at any level by three-way ANOVA. TTRF was greater for individuals younger than 1 year and for samples from females, yet TTRF and pH did not co-vary with expression of any genes.

Based on medical histories for inflammation, 25 out of 58 individuals experienced some form of inflammation proximate to death, having succumbed to viral or bacterial infections, myocarditis (of which 80% or more is caused by a viral infection), fever, or asthma. If there were no indications of inflammation or use of non-steroidal anti-inflammatory drugs or antibiotics, then individuals were classified as “no inflammation,” of which there were 33, most of whom perished by car or other sudden accident.

We were able to characterize expression of all targeted genes except *EP1*, which we could not detect. Graphing each gene, including reference genes, against PMI also revealed that generally RNA did not degrade with PMI. This was true for RNA of particularly sensitive enzymes such as *CYP19A1*, *PTGS1* (COX-1), *PTGS2*, *MGLL* (monoacylglycerase lipase or MAG lipase), and *PGES1* (microsomal PGE synthase-1), which also did not co-vary with brain pH, or TTRF.

**Fig. 1:**
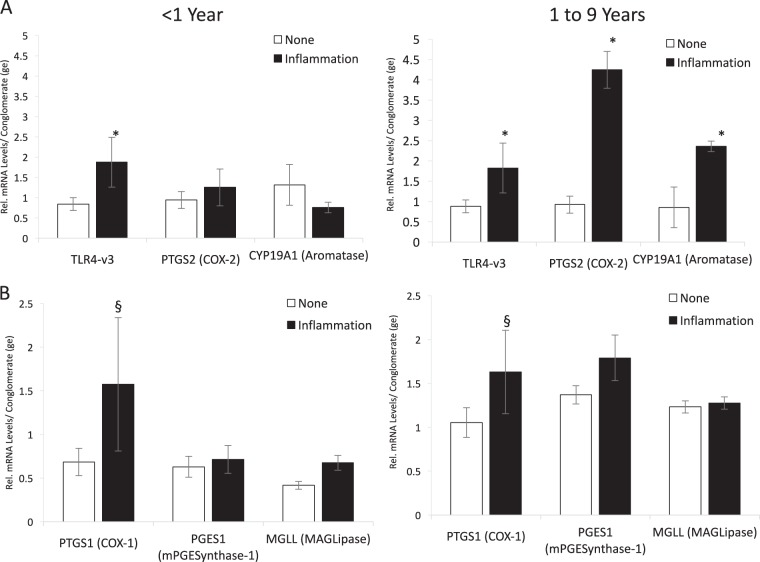
Gene expression profile of the PGE2-E2 pathway in developing cerebellum. Subjects were divided into those (**a**) under 1 year of age and **b** those older, up to 9 years and categorized as experiencing inflammation or none at the time of death (see text for details and rational). Categorization as inflamed was confirmed by quantification of *TLR4* mRNA, which was significantly higher (*F*_inflam_ = 8.43, *p = 0.005). Levels of mRNA were quantified in cerebellar samples for genes associated with prostaglandin and estradiol synthesis. *PTGS1* mRNA was marginally increased in subjects experiencing inflammation regardless of age (*F*_inflam_ = 6.66, *p* = 0.077). *PTGS1* and *CYP19a* were significantly increased in inflamed subjects over 1 year of age (*PTGS2*: *F*_age × inflam_ = 6.73, *p = 0.013, *CYP19a*: *F*_age ×inflam_ = 4.36, *p = 0.043). PGE2 prostaglandin E2, TLR4 Toll-like receptor 4, mRNA messenger RNA

Up-regulation of *TLR4* mRNA is a reliable indicator of inflammation^25,29^. Expression of TLR4 variant 3 was significantly higher in cerebellums from children categorized as experiencing inflammation regardless of their age or sex (*F*_inflam_ = 8.43, *p* = 0.005), validating our classification based on medical records (Fig. 1a, b). The other two TLR4 variants (TLR4-v1 and TLR4-v4) also responded to inflammation, albeit with more complex effects. TLR4v1 expression exhibited a three-way interaction between inflammation, age, and sex (Fig. S1A—*F*_3-way_ = 12.37, *p* = 0.019). TLR4v4 expression was significantly influenced by inflammation, but also independently exhibited a two-way interaction between sex and age (Fig. S1B—*F*_inflam_ = 15.33, *p* = 0.006, *F*_sex × age_ = 12.54, *p* = 0.014). In both instances, the significant interaction appears due to elevated TLR4 mRNA in females under 1 year of age with inflammation. However, given the small sample size (*n* = 4), this observation should be interpreted with caution.

Almost one-third of our subjects were infants that died in the first year of life; however, only three of the 21 died in the second half of the year. Given the natural break in the data set, we treated the 1-year boundary in age as a distinct factor. The mRNAs of only a few of the genes we examined were affected by inflammation in individuals younger than 1 year of age: (1) TLR4v3 (discussed above), (2) *PTGS1* mRNA, which trended higher with inflammation regardless of age (*F*_inflam_ = 6.66, ^§^*p* = 0.077), and (3) the PGE2 receptor PTGER4, which trended lower with inflammation in individuals younger than 1 year of age (*F*_age × inflam._ = 3.15, ^§^*p* = 0.085).

In contrast, in subjects aged 1–9 years experiencing inflammation at the time of death, there was a significant increase in mRNA levels for *PTGS2*, and *CYP19a* compared to individuals not experiencing inflammation (*PTGS2*: *F*_age × inflam = _6.73, *p* = 0.013; *CYP19a*: *F*_age × inflam_ = 4.36, *p* = 0.043, Fig. 1a, b).

**Fig. 2:**
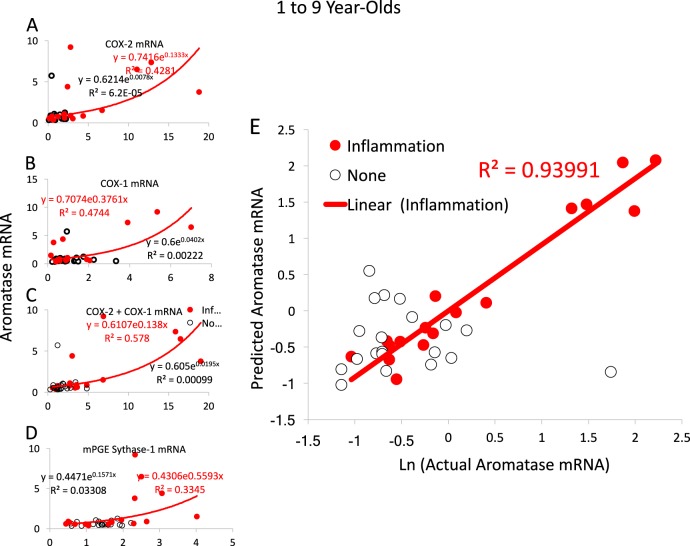
Correlations and linear modeling of CYP19a with PGE2 synthesizing enzymes. **a** mRNA levels for PTGS1 and **b** PTGS2 (COX-1 and COX-2, respectively) predict 47 and 42% of the variance in *CYP19a* mRNA expression after exponential regression of the subjects with inflammation older than 1 year. **c** When combined into a total *PTGS* mRNA measure, 57.8% of the variability in *CYP19a* mRNA was predicted. **d** *PGES1* (PGE synthase) mRNA levels predict 33.5% of the variance in *CYP19a* mRNA levels in individuals aged 1 to 9 years with inflammation. **e** Automatic linear modeling confirmed the effects of PGE2 signaling and inflammation on *CYP19a—*94% of the variance in mRNA expression could be predicted by total *PTGS*, *PGES1*, *TLR4v4*, *TLR4v1*, *CALB1*, and *ERS2* expression in individuals experiencing inflammation at the time of death. PGE2 prostaglandin E2, COX cyclooxygenase, mRNA messenger RNA

To determine whether there is a relationship between expression of inflammation-associated genes and estradiol-associated genes, we began with a simple regression of *CYP19a* mRNA levels against expression of each candidate gene individually. For *PTGS1*, *PTGS2*, and *PGES1*, we found significant positive correlation with *CYP19a* mRNA (Fig. 2a, b, d). *PTGS1* and *PTGS2*, respectively, accounted for 47 and 42% of the variance in *CYP19a* mRNA expression after exponential regression of the subjects with inflammation older than 1 year. The same regression in individuals without inflammation accounted for <0.01% of the variance. Combining *PTGS1* and *PTGS2* into a measure of total COX mRNA accounted for 57.8% of the variability in *CYP19a* mRNA in individuals with inflammation (Fig. 2c). Likewise, PGES-1 mRNA could account for a significant 33.5% of the variance in *CYP19a* mRNA levels (Fig. 2d).

To assure these a priori correlations do not represent an unseen bias or that other genes might better correlate with aromatase expression, we performed an automatic step-wise regression analysis of mRNA levels for all 20 genes we measured (listed in Table S1). Since *PTGS1* and *PTGS2* serve the same function, making PGH2, we hamstrung the automatic linear modeling by inputting total *PTGS* mRNA levels, weighting each *PTGS* relative to the other’s cycle threshold. Ninety-four percent of the variability of aromatase expression in cerebellar tissue from individuals with inflammation aged 1 year and older was accounted for by exponentially regressing total COX (coef 0.201, *p* = 0.001), TLR4v4 (coef 1.10, *p* = 0.001), TLR4v1 (coef −1.12, *p* = 0.001), CALB1 (coef −0.437, *p* = 0.007), *PGES1* (coef 0.253, *p* = 0.011), and *ERS2* expression (coef 0.05, *p* = 0.012) (Fig. 2e; for illustration Fig. S2 shows individual correlations of CYP19a with mRNA for *PGES1* (*R*^2^ = 33.5%), *TLR4v1* (*R*^2^ = 36.6%), *TLR4v4* (*R*^2^ = 38.5%), *ESR2* (*R*^2^ = 6.8%), CALB1 (*R*^2^ = 36.0%)). By comparison, the same analysis done in subjects without inflammation older than 1 year of age found no significant correlations and no predictive power (Fig. 2e).

**Fig. 3:**
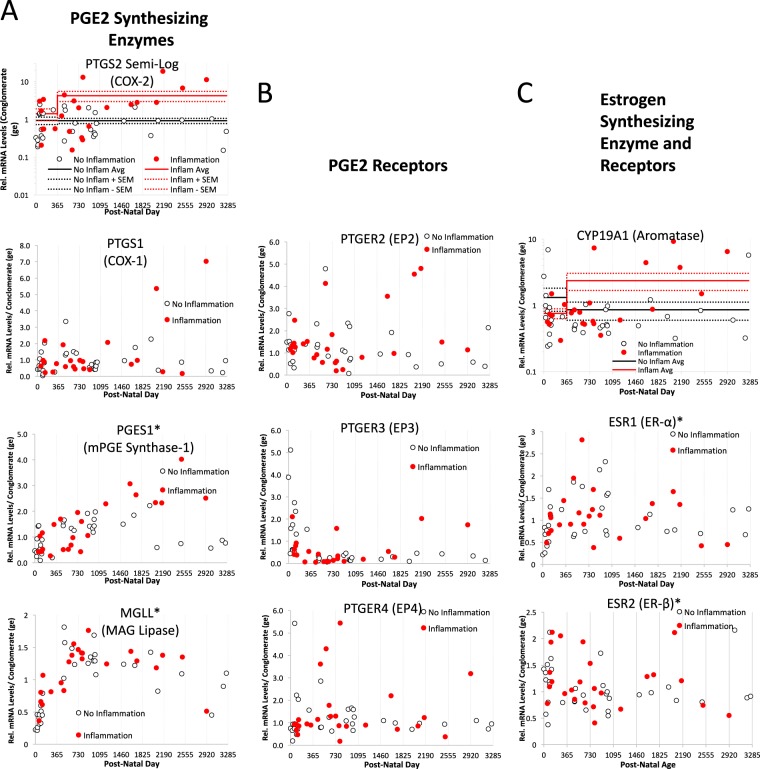
Developmental profile of the PGE2-E2 pathway in cerebellum. Plotting of individual mRNA levels for each gene across age of subjects reveals that *MGLL* (*F_age_ = 43.32, *p* < 0.001), *PGES1* (*F_age_ = 14.21, *p* < 0.001), *ESR1* (*F_age_ = 4.63, *p* = 0.03), and *ESR2* (*F_age_ = 12.21, *p* < 0.001) changed significantly with age, increasing as individuals got older, with the exception of ESR2 which declined. RNA for other genes measured did not change across development. PGE2 prostaglandin E2, mRNA messenger RNA, ESR2 estrogen receptor 2

An important question was why inflammation did not increase *CYP19a* or *PGHS2* mRNA in children under 1 year of age. In our studies of the rat model, we discovered an intrinsic gene expression profile that determined a sensitive window of development by dynamic turning on and off of particular genes at specific ages.^24^ Attempts at a similar analysis in humans is challenged by compromised health of most subjects and a non-random age distribution skewed towards birth. The relative mRNA levels for genes relevant to PGE2 and estradiol synthesis and function are plotted across age in days in Fig. 3. The majority of gene expression profiles did not vary systematically between individuals younger and older than 1 year of age, with the exception of *MGLL*, *PGES1*, and *ESR1*, all of which increased with age (*MGLL*: *F*_age_ = 43.32, *p* < 0.001; *PGES1:* *F*_age_ = 14.21, *p* < 0.001; *ESR1*: *F*_age_ = 4.63, *p* = 0.03), and *ESR2 and CALB1* that decreased with age (*ESR2*: *F*_age_ = 12.21, *p* < 0.001; *CALB1*: *F*_age_ = 9.35, *p* = 0.004). However, these changes occur independent of any effects on aromatase. Given the power of the automatic linear modeling in predicting aromatase expression in individuals over 1 year of age with inflammation, we asked whether the expression of the 20 genes could also predict aromatase expression across all of development in all 58 individuals, regardless of inflammation. Fifty four percent of the variance in exponentially regressed aromatase mRNA could be accounted for by the PGE2 receptors EP3 (coef 0.597, *p* = 0.001) and EP4 (coef 0.37, *p* = 0.001) and the PGE2 synthetic enzyme, membrane-associated PGE (mPGE) synthase (coef 0.624, *p* = 0.001), suggesting that prostaglandins promote estradiol synthesis in the developing human cerebellum in health and disease.

## Discussion

We recently described a novel sensitive period in cerebellar development in the rat in which peripheral inflammation induces central PGE2 production that in turn stimulates the estradiol-synthesizing enzyme, aromatase, resulting in elevated cerebellar estradiol^22,23^. We here sought to determine whether an analogous pathway exists in the developing human cerebellum. We predicted that the enzymes producing PGE2 would be increased in the cerebellum of infants and children experiencing infection or inflammation at the time of death. We further predicted that aromatase would also increase in these individuals, and the expression of the two should correlate. As expected, we found that COX-2 mRNA levels were elevated in individuals with inflammation, but unexpectedly only when over 1 year of age. Aromatase expression followed the same pattern, increasing in children with inflammation over 1 year old but not before. COX-1 mRNA trended higher with inflammation, regardless of age. There was no effect of gender on these measures, which is consistent with the lack of any influence of sex we observed in the rat.

### Inflammation in postnatal human cerebellum correlates with increased aromatase mRNA

After 1 year of age a significant positive correlation between the mRNA levels for the COX enzymes and aromatase was observed in individuals with inflammation. When the number of parameters was expanded, a remarkable 94% of biological variability in aromatase expression could be controlled for by modeling mRNA levels for total COX (COX-1 and COX-2 combined), mPGE synthase, TLR4v1 and TLR4v4, ER-β, and calbindin. While correlations do not imbue causation, the results support the existence of a human cerebellar pathway in which PGE2 signaling regulates cerebellar aromatase expression in early childhood.

### The effect of inflammation on aromatase does not appear until after 1 year of age

There were two notable differences between our observations in the rat and in human subjects. First is the lack of an association between inflammation and estradiol production in newborn infants. Almost a third of our subjects died at or shortly after birth, with an additional few perishing within 1 year of age. It is possible that the parameters surrounding death in these individuals is distinctly different from that of children that have experienced a healthy postnatal period. Alternatively, the lack of an association between inflammatory parameters and aromatase expression in individuals immediately after birth may have its origins in the very low levels of PGE synthase we detected in the cerebellum. Up to 54% of aromatase expression could be accounted for by modeling the expression of PGE synthase, along with EP3 and EP4, at all ages. We conclude from this that PGE signaling does not merely control aromatase expression during inflammation but also across the full range of development.

In the rat model, we found a naturally occurring peak in Cyp19a and ERS1 expression during the second postnatal week, the height of the sensitive period, but by the third postnatal week those levels have dropped precipitously^24^. The relationship between developmental events in the rodent vs. human brains is complicated by choice of endpoint and brain region. An informal “meta-analyses” of close to 40 manuscripts examining the timing of glial and neural genesis, synaptogenesis, and oligodendrocyte maturation in humans and rodents concluded that the period from PN10–PN20 in the rodent is equivalent to 2–3 years in the human^30^, which places our findings in the rodent at the post-1 year stage. Others argue, based on cortical development, that the 2nd postnatal week in the rat is closer to a 2nd–3rd trimester human^31^. Interestingly, a comparison of gene expression in multiple brain regions across development suggests that the cerebellum shows the least correlation between postnatal mouse and human and that it is birth that is the singular event for this brain region in both species, likely due to its central role in motor control and sensory processing^28^.

Prostaglandins have been previously linked to estradiol production in the cerebellum as well as the periphery. In both cases, the stimulation of aromatase by PGE2 requires activation of PKA, which is achieved via its adenylate cyclase-linked EP receptors^32^. The stimulation of estradiol synthesis has been most strongly tied to the PGE2 receptor EP4^33,34^. We found that PGE2 induction of aromatase in the rodent cerebellum is also PKA dependent^24^. The automatic linear modeling done here suggests that the human cerebellum is similar, with at least 54% of aromatase expression dependent upon the relative expression of EP3 and EP4, as well as the final enzyme in PGE2 synthesis. If neither PGE2 nor EP4 are present, there should be no induction of aromatase. By contrast, EP3 (*PGER3*) mRNA levels were high in the first year and then dropped. EP3 is among the most complex of the prostanoid receptors with multiple splice variants, divergent signal transduction pathways, and potentially opposing effects^35^. EP3 frequently counteracts EP4 because of its inhibition of cAMP production^36^. It is possible that EP3 serves as a negative transducer of PGE2 by preventing an induction of aromatase at a time when excessive estradiol would be deleterious to the developing cerebellum. Moreover, while the COX-2 enzyme was present during the first year, it was not induced by inflammation, further suggesting a protective mechanism dampening inflammatory mediators within the cerebellum.

The second notable difference is that, unlike in the rat, we did not see a closing of the sensitive period in children up to age 9 years. The number of human subjects available diminished with increasing age, as did the incidence of inflammation, and thus we may have been underpowered to detect a dissociation between PGE2 signaling and induction of aromatase later in life, as seen in the rat. Alternatively, the sensitive period in humans may be much longer than that of the rodent consequent to the far greater complexity of human brain development. It is worth noting that the relationship we observed between inflammation and Cyp19a expression spanned at least 8 years in early life, a time when the cerebellum is expanding in both size and complexity. That the correlation persisted attests to the robustness of the regulatory control between prostaglandin production and estradiol synthesis in the human brain.

### Inflammation and risk of ASD and schizophrenia

Inflammation during the first and second trimester of pregnancy is among the strongest environmental factors that correlate with later diagnosis of schizophrenia or ASD in the offspring^14,37^, but the overwhelming majority of pregnancies in which inflammation occurs do not result in offspring with later neuropsychiatric diagnosis. Unraveling which children are at risk is a major unmet goal. Importantly, the deleterious consequences of in utero inflammation do not manifest for years, when the child is older and critical brain functions come online. There has yet to be a direct link between changes in the brain at the time of inflammation in utero and later diagnosis of ASD or schizophrenia. This may be a simple artifact of technical limitations, but could also reflect that the immature brain is incapable of directly incorporating the impact of the insult at that time. Instead, it is possible that the ongoing inflammation intersects with a later sensitive period, and signaling cascades therein, to alter the developmental trajectory. Recent work reveals the enduring effects of in utero inflammation in non-human primate offspring which last up to 4 years^38^.

In addition to in utero inflammation, some have attributed the dramatic increase in ASD diagnosis since the 1980s to overall increases in inflammation associated with western civilization and the attendant changes in diet, pollution, stress, and so on, and this includes exposures to infants and very young children^39^. There is also a growing appreciation that developmental delays in social and other skills may manifest years before an autism diagnosis is made^40^, especially in girls^41,42^, illustrating we still have much to learn about this complex neurodevelopment disorder. One compelling suggestion is the increased use of acetaminophen as an analgesic in children, particularly following the discovery of aspirin-induced Reye’s syndrome in the 1980s. Acetaminophen blocks prostaglandin production, as do nonsteroidal anti-inflammatory drugs, but also has additional potentially toxic effects related to oxidative stress and may actually increase inflammation rather than decrease it (reviewed in ref. ^39^). Inextricably, there is also a subset of children with ASD whose symptoms improve during fever^43^ and local production of estradiol, a well-established trophic factor in developing brain^44^, may be a contributing variable. Understanding the cellular mechanisms controlling sensitive periods in brain development, including those that may occur postnatally, will be an essential piece of the neuropsychiatric puzzle. The data presented here suggest a sensitive period beginning after 1 year of age in humans in which inflammation is linked to endogenous estradiol production in the cerebellum. Extrapolating from basic research in rodents in which social behavior was impacted, it is likely that an imbalance in steroidogenesis in the developing brain is a contributing factor to dysregulation and variation in the onset of socially regressive symptoms, which could in part underpin the ultimate risk for and severity of the neuropsychiatric disorder. Finding and characterizing sensitive periods in brain development is essential to unraveling the myriad of complex internal and external variables that contribute to health and disease.

## Supplementary information

Table S1

Figure S1

Figure S2

Figure S3

Supplemental legends
