# Supplementary material for: Evidence that inflammation promotes estradiol synthesis in human cerebellum during early childhood
Source: Transl Psychiatry. 2019 Jan 31;9:58. doi: 10.1038/s41398-018-0363-8 (PMC6355799; doi:10.1038/s41398-018-0363-8)
Supplement: Supplementary file 1 — Table S1 [file 41398_2018_363_MOESM1_ESM.docx]

**Supplemental Table 1:** *Primer Sequences for human genes*

| **Human Transcript cDNA Primers** | | | | | |  |
| --- | --- | --- | --- | --- | --- | --- |
| PTGS1 (COX1) | NM_000962.3  NM_080591.2  NM_001271164.1  NM_001271165.1  NM_001271166.1  NM_001271367.1  NM_001271368.1 | F 5’-CAATGAGTACCGCAAGAGGTTTG; 1466 bp  R 5’-GTAGAACTCCAACGCATCAATGTC; 1589 bp | 125 | 60 | 80 | |
| PTGS2 (COX2) | NM_000963.3 | F 5’-AACATTCCCTTCCTTCGAAATG; 405 bp  R 5’-TCCCAGCTTTTGTAGCCATAGTC; 514 bp | 110 | 60 | 76.8-77 | |
| PGES1 (Microsomal PGE-synthase-1) | NM_004878.4 | F 5’-CTTTGCCAACCCCGAGGAT; 182 bp  R 5’-GGTTAGGACCCAGAAAGGAGTAGAC; 339 bp | 158 | 60 | 84.5 | |
| Mgll (MAG Lipase) | NM_007283.6  NM_001003794.2 | F 5’-CGTGCTCTCTCGGAATAAGAC; 1140 bp  R 5’-AGTTGGATGCCGAAGCAC; 1233 bp | 94 | 60 | 80.5 | |
| PTGER2 (EP2) | NM_000956.3 | F 5’-AAAATGGGACCTCCAAGCTCTT; 1133 bp  R 5’-CAGGAAGTTTGTGTTGCATCTTGT; 1288 bp | 156 | 60 | 77.87 | |
| PTGER3  (EP3) | NR_028294.1  NR_028292.1  NR_028293.1  NM_198714.1  NM198715.2  NM_198716.1  NM_198717.1  NM198718.1  NM_198719.1  NM_001126044.1 | F 5’-CCGCTCCTGATAATGATGTTGA; 1120 bp  R 5’-GCAGGTAAACCCAAGGATCCA; 1274 bp | 155 | 60 | 78.8-79 | |
| PTGER4 (EP4) | NM_000958.2 | F 5’-AGGGCTATCATCATCCTACAACTCA;’ 2150 bp  R 5’-TAGGTCTTCGCAGCCATCAAG; 2313 bp | 164 | 60 | 81.5 | |
| CYP19A1  (Aromatase) | NM_000103.3  NM_031226.2 | F 5’-CTTTGCCACTGAGTTGATTTTAGC; 973 bp  R 5’-TTAGGGTGCTTTGCAATGAGAA; 1122 bp | 150 | 60 | 79 | |
| ESR1  (ERα) | NM_000125.3  NM_001122740.1  NM_001122741.1  NM_001122742.1  NM_001291230.1  NM_001291241.1 | F 5’-TCCAGCACCCTGAAGTCTCT; 1621 bp  R 5’-GATGTGGGAGAGGATGAGGA; 1778 bp | 158 | 60 | 82.9 | |
| ESR2 (ERβ) | NM_001437.2  NM_001040275.1  NM_001214902.1  NM_001271876.1  NM_001271877.1  NR_072496.1  NR_073497.1  NM_001291712.1  NM_001291723.1 | F 5’-ACATCTGTATGCGGAACCTCAAAA; 756 bp  R 5’-CCCTCTTTGAACCTGGACCAGTA; 901 bp | 146 | 60 | 80.3 | |
| TLR4-v1 | NM_138554.4 | F: 5’-TCACAGAAGCAGTGAGGATGAT; 271 bp  R: 5’-AAGTAATATTAGGAACCACCTCCA; 414 bp | 174 | 60 | 84.2-84.5 | |
| TLR4-v3 | NM_003266.2 | F: 5’-TTAGGAACCACCTCCGTGATAA; 394bp  R 5’-TTGGCCCTAAACCACACAGAA; 522 bp | 129 | 60 | 81.3 | |
| TLR4-v4 | NM_138557 | F 5’-TCACAGAAGCAGTGAGGATGAT; 271 bp  R 5’-ATTTCACACCTCCACGCAGG; 400 bp | 130 | 60 | 84 | |
| Rlp13A | NM_012423.3  NM_001270491.1  NR_0730-24.1 | F 5’-TGTTTGACGGCATCCCAC; 387 bp  R 5’- CTGTCACTGCCTGGTACTTC; 536 bp | 150 | 56-58 | 83.9 | |
| WHYAZ | NM_003406.3  NM_145690.2  NM_001135699.1  NM_001135700.1  NM_001135701.1  NM_001135702.1 | F 5’-AAGCCATTGCTGAACTTGATACATTAA; 805 bp  R 5’-CAGCTTCGTCTCCTTGGGTATCC; 924 bp | 120 | 58-60.2 | 77 | |
| TATA Box Protein | NM_003194.4  NM_001172085.1 | F 5’-CAGGAGCCAAGAGTGAAGAACA; 944 bp  R 5’-TGGAAAACCCAACTTCTGTACAAC; 1020 bp | 77 | 56-58 | 76.4-76.6 | |
