# Supplementary figures and images for: Evidence that inflammation promotes estradiol synthesis in human cerebellum during early childhood
Source: Transl Psychiatry. 2019 Jan 31;9:58. doi: 10.1038/s41398-018-0363-8 (PMC6355799; doi:10.1038/s41398-018-0363-8)

A

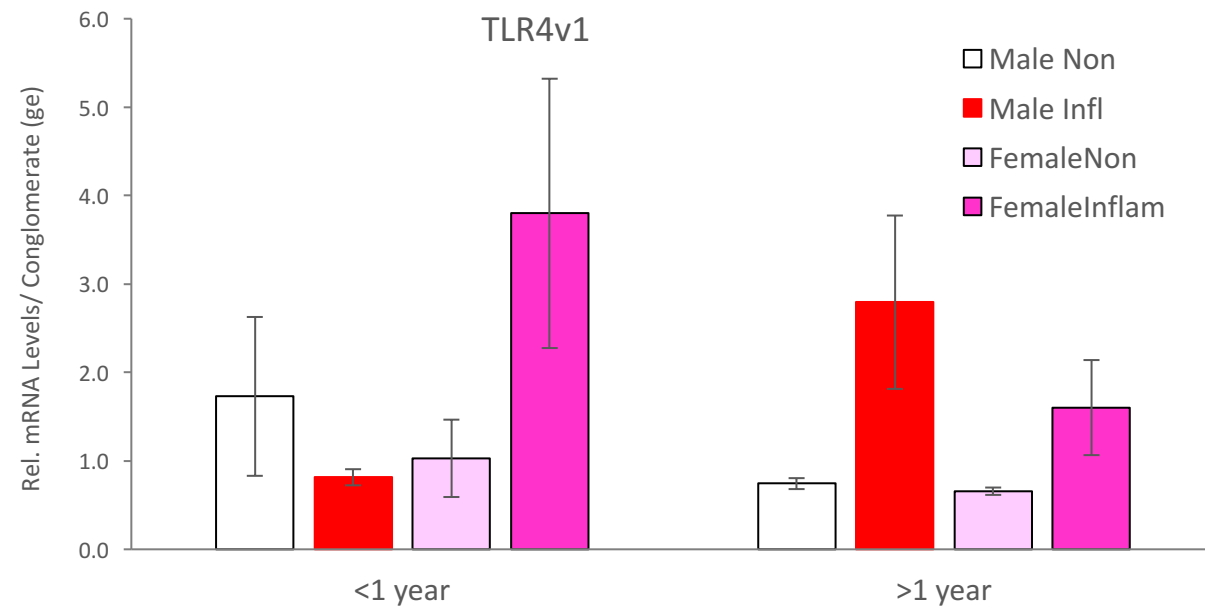

B

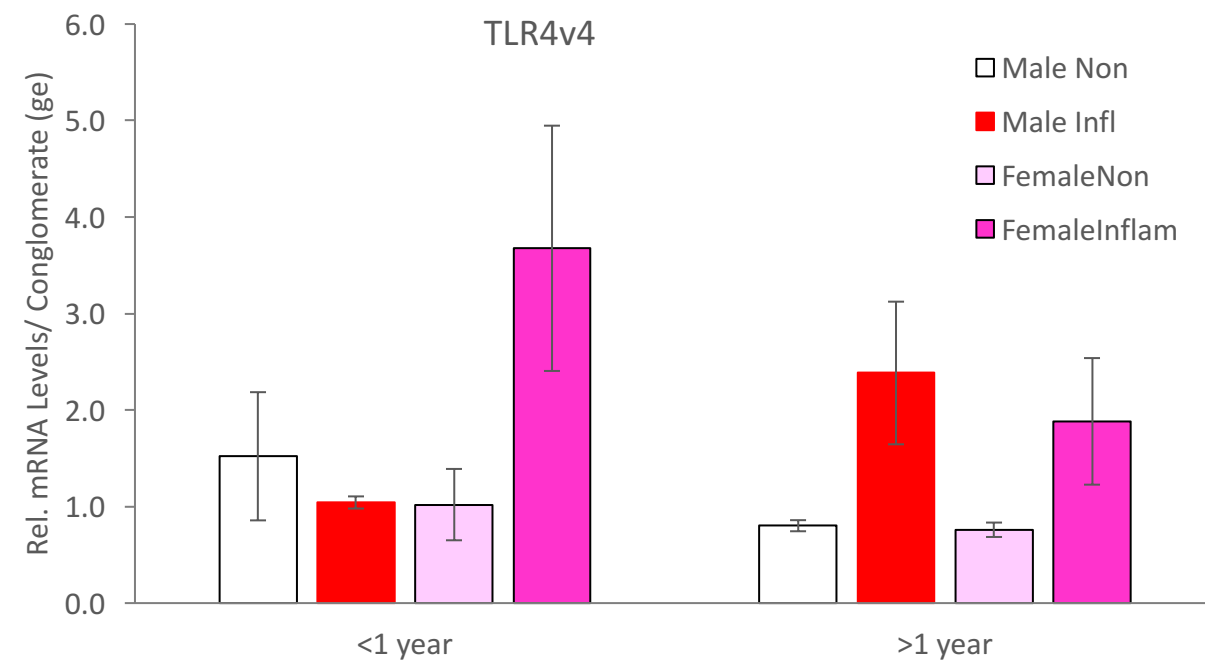

Supplement: Supplementary file 2 — Figure S1 [file 41398_2018_363_MOESM2_ESM.pdf]

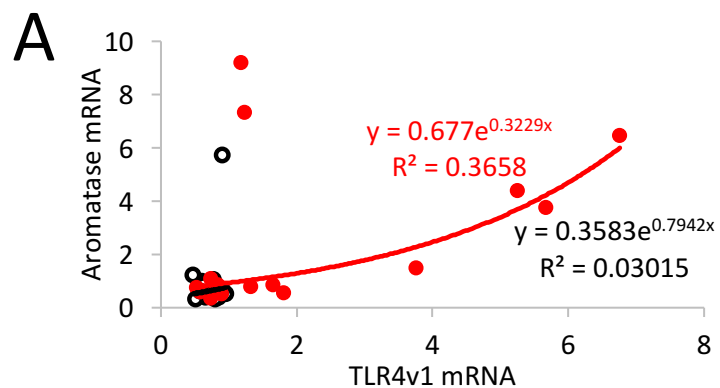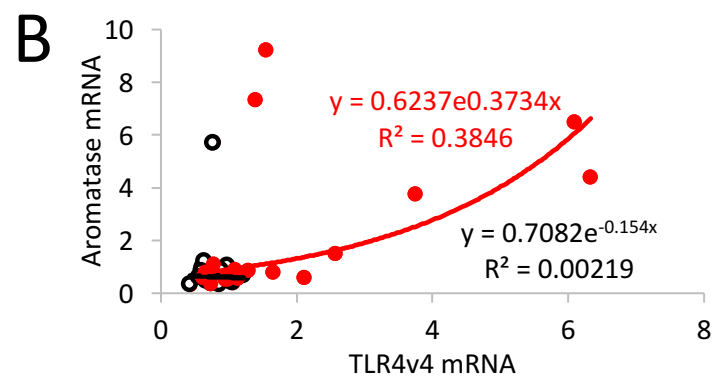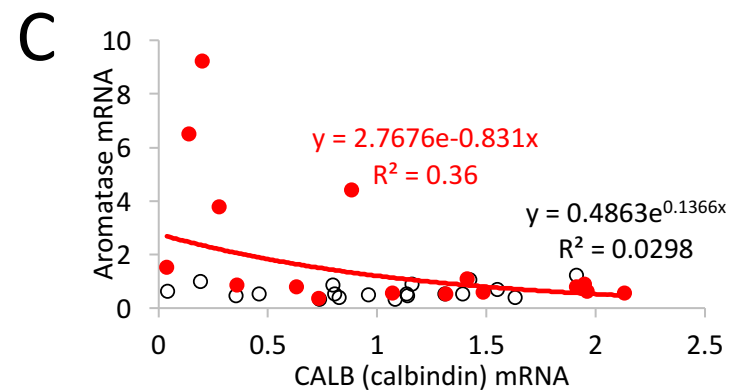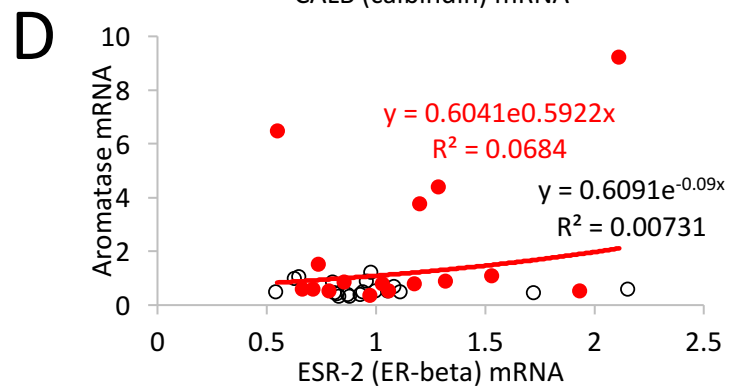

Supplement: Supplementary file 3 — Figure S2 [file 41398_2018_363_MOESM3_ESM.pdf]

# All Ages

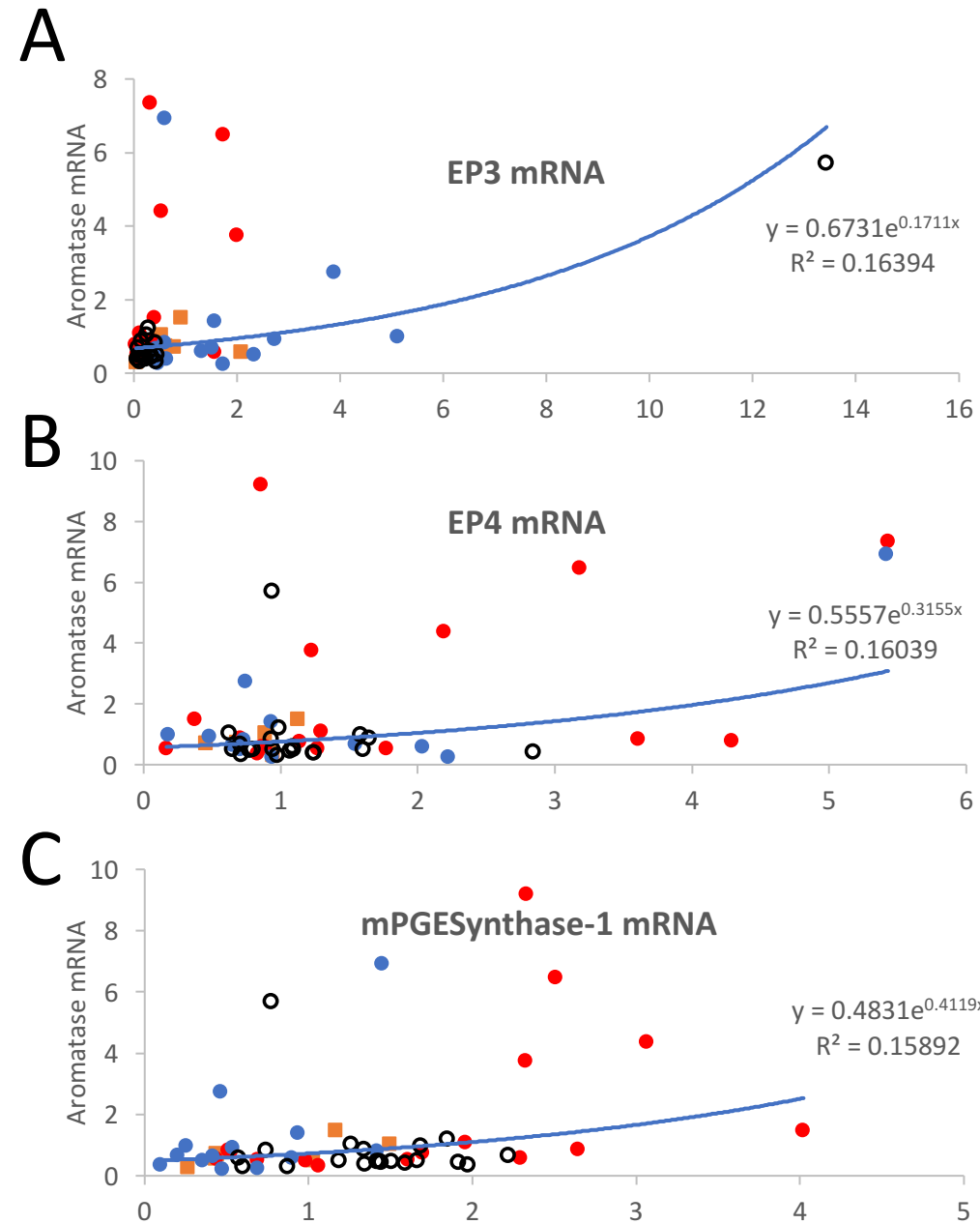

**D**

## Predicted Aromatase mRNA From Combined Model

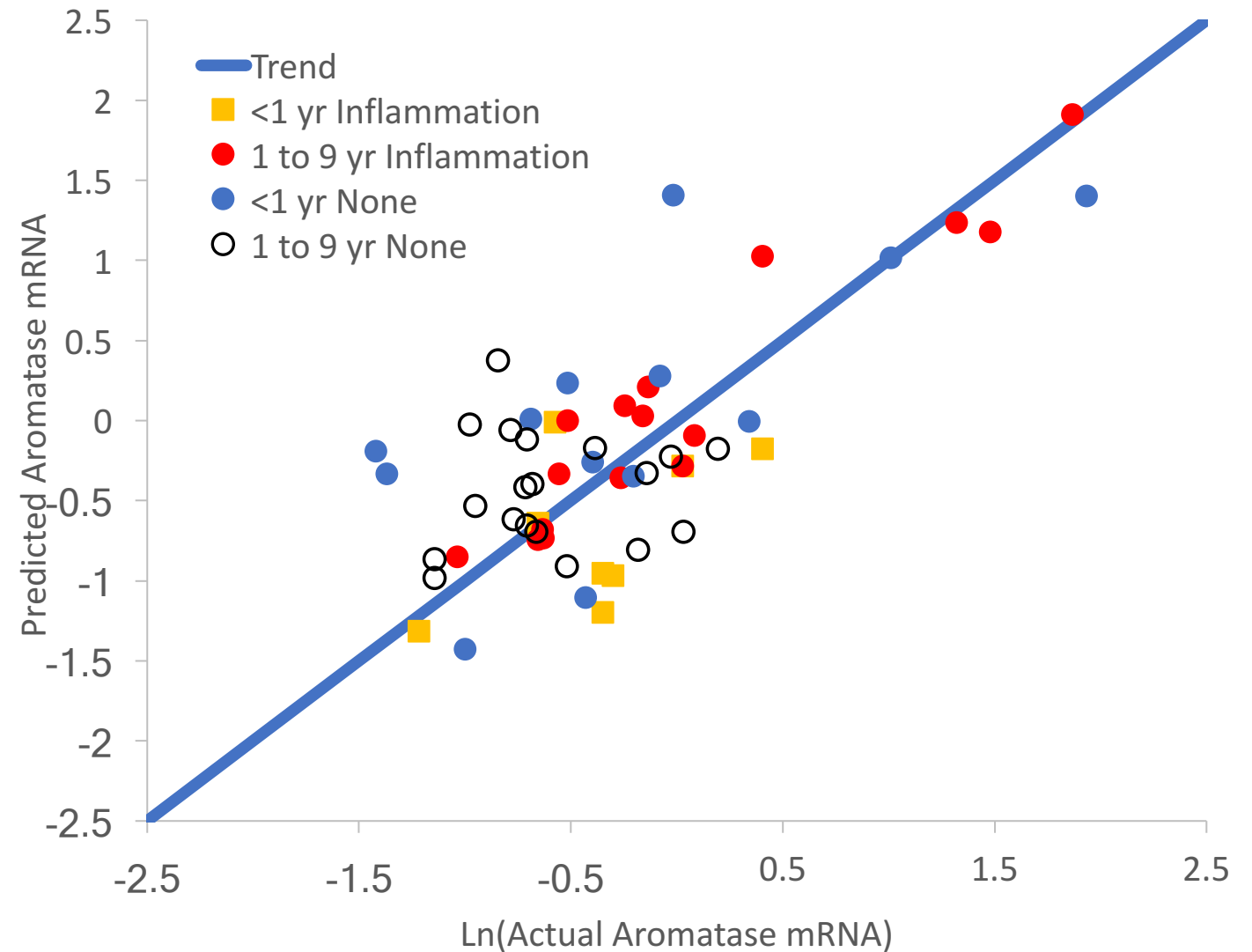

Supplement: Supplementary file 4 — Figure S3 [file 41398_2018_363_MOESM4_ESM.pdf]
