# Supplementary material for: Evidence that inflammation promotes estradiol synthesis in human cerebellum during early childhood
Source: Transl Psychiatry. 2019 Jan 31;9:58. doi: 10.1038/s41398-018-0363-8 (PMC6355799; doi:10.1038/s41398-018-0363-8)
Supplement: Supplementary file 5 — Supplemental legends [file 41398_2018_363_MOESM5_ESM.docx]

**Supplementary Figure 1: Interaction between age, gender, and inflammation affects TLR4v1 (A) and TLR4v4 (B) expression.** TLR4v1 and TLR4v4 mRNA levels increased with inflammation in both males and females older than one year of age and also in females younger than one year of age. Yet, in males less than one year of age, TLR4v1 and TLR4v4 mRNA levels were unchanged with inflammation.

**Supplementary Figure 2: Correlations between PGE2 synthesis and aromatase expression arise with age and inflammation.** The levels of mRNA for the following genes also individually exponentially correlate with cerebellar aromatase mRNA levels in the individuals over 1 year of age experiencing inflammation but not in individuals without inflammation: (A) TLR4v1 (R^2^=0.366), (B) TLR4v4 (R^2^=0.385), (C) CALB (calbindin: R^2^=0.36), and (D) ESR-2 (ER-beta: R^2^=0.0684). There was no correlation between the same genes and aromatase mRNA in individuals not experiencing inflammation.

**Supplementary Figure 3: Inclusion of all subjects in correlation of mRNA for receptors for PGE2 and its terminal synthase with aromatase expression.** When ages and inflammatory states are combined, (A) EP3 (R^2^=0.164), (B) EP4 (R^2^=0.160), and (C) mPGESynthase-1 (R^2^=0.159) mRNA levels correlate exponentially with aromatase mRNA levels. (D) But, when combined into an automatic model with mRNA levels for all the other genes, expression of these genes and not the others could account for 54.1% of the variability in aromatase expression for the entire data set. (Step-wise automatic modeling of natural log of aromatase (exponential regression), criteria for including gene in model p<0.05.)
